# Anterior cervical discectomy without fusion for a symptomatic cervical disk herniation

**DOI:** 10.1007/s00701-017-3189-x

**Published:** 2017-04-27

**Authors:** Judith D. de Rooij, Pravesh S. Gadjradj, John S. Soria van Hoeve, Biswadjiet S. Harhangi

**Affiliations:** 1 Department of Neurosurgery, Erasmus MC: University Medical Center Rotterdam, S-Gravendijkwal 230 HS-205, 3015 CE Rotterdam, The Netherlands; 2 Department of Pain Medicine, Erasmus MC: University Medical Center Rotterdam, S-Gravendijkwal 230 HS-205, 3015 CE Rotterdam, The Netherlands; 3 Department of Physical Therapy, Erasmus MC: University Medical Center Rotterdam, S-Gravendijkwal 230 HS-205, 3015 CE Rotterdam, The Netherlands; 4 Department of Neurosurgery, Leiden University Medical Center, Leiden, The Netherlands; 5 Department of Health Sciences, Vu University Amsterdam, Amsterdam, The Netherlands

**Keywords:** Anterior cervical discectomy, Cervical radiculopathy, Disk herniation

## Abstract

**Background:**

Cervical radiculopathy is characterized by dysfunction of the nerve root usually caused by a cervical disk herniation. The most important symptom is pain, radiating from the neck to the arm. When conservative treatment fails, surgical treatment is indicated to relieve symptoms. During the last decades, multiple fusion techniques have been developed, although without clinical evidence for added value of fusion over non-fusion.

**Methods:**

The surgical procedure of anterior cervical discectomy without fusion is performed step by step, leading to removal of the entire intervertebral disk.

**Conclusion:**

Anterior cervical discectomy without fusion is a safe and effective treatment for cervical disk herniation.

**Electronic supplementary material:**

The online version of this article (doi:10.1007/s00701-017-3189-x) contains supplementary material, which is available to authorized users.

## Relevant surgical anatomy

**Fig. 1:**
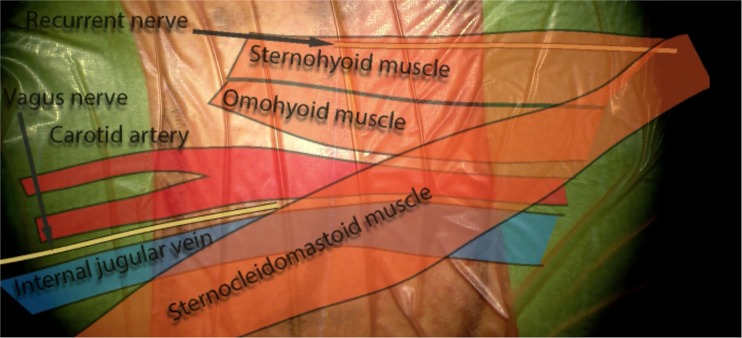
Surgical anatomy of the anterior neck

Under the platysma, the deep cervical fascia invests the deeper layers of the neck (Fig. 1). This area can be further divided into the superficial, middle and deep layers. The superficial layer covers the sternocleidomastoid muscle (SCM), while the middle layer includes the esophagus, trachea, thyroid and strap muscles such as the sternohyoid and the omohyoid muscles. The deep layer surrounds the cervical vertebrae with the paraspinal muscles. The carotid artery, internal jugular vein and vagus nerve are covered within the carotid sheath. Both the right and left recurrent laryngeal nerves (RLN) branch off the nervus vagus and follow the carotid artery in the neck before they loop. The right RLN, however, loops under the right subclavian artery, while the left RLN loops under the aortic arch.

## Preoperative workup

Correct upper airway management for cervical spine surgery is important for successful anesthesia. Minimal movement of the neck is warranted during intubation to prevent spinal cord injury [4]. After general anesthesia the patient is positioned supine with the head in light extension. Intraoperative neuromonitoring is only used if there is a significant spinal cord compression. Recent research showed no difference in the risk of neurological injury when performing an anterior cervical discectomy (ACD) with or without intraoperative neuromonitoring [1].

**Fig. 2:**
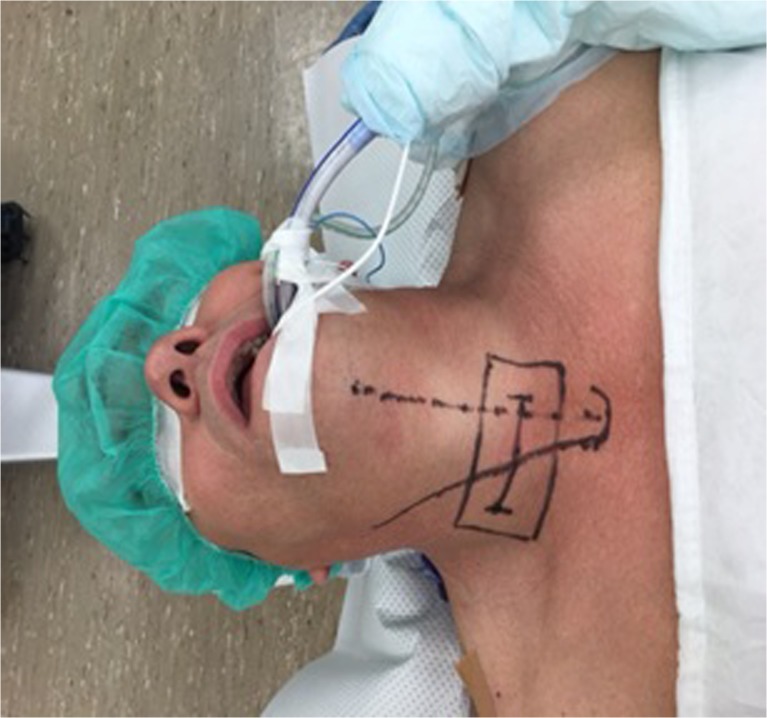
Positioning and marking of the midline, jugulum and sternocleidomastoid muscle

**Fig. 3:**
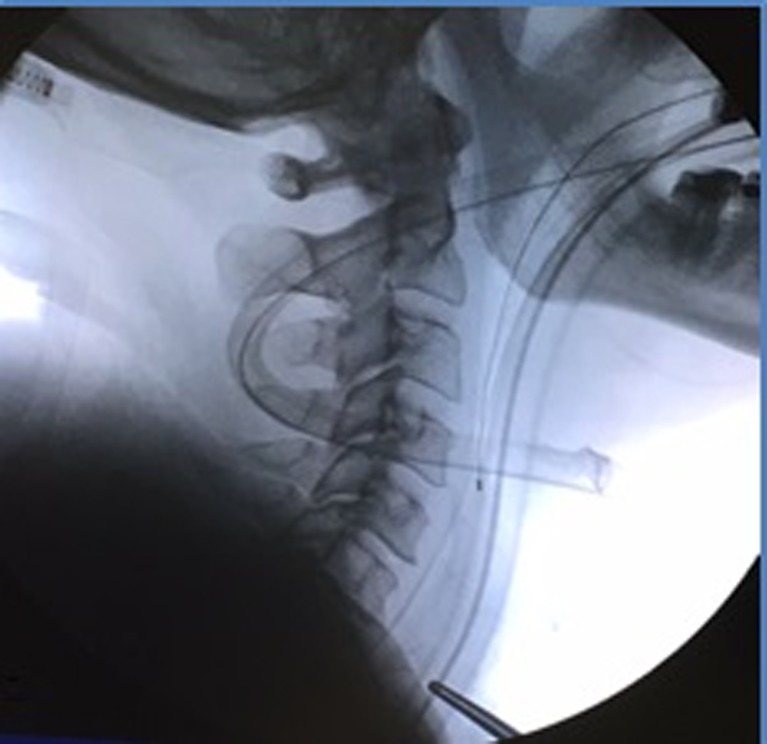
Identifying the appropriate surgical level with intraoperative fluoroscopy

After positioning the midline, the jugulum and SCM are marked (Fig. 2), and the appropriate surgical level is identified using fluoroscopy (Fig. 3). Fluoroscopic exposure of the lower cervical vertebrae may be hindered by the shoulders, which is solved by retracting the shoulders caudally. At higher levels the mandibula is rotated to the contralateral side to facilitate surgical exposure. The authors prefer to enter the disk from the contralateral side of the disk herniation to have a good view of the exiting nerve root.

## Surgical technique

The level of disk herniation is identified using fluoroscopy. The angle of approach should be in the extension of the disk space, perpendicular to the anterior longitudinal ligament (ALL).

A 5-cm skin crease incision is made on the right or left side of the neck, and the platysma is identified in the superficial fascia. After transecting the platysma toward the pretracheal fascia a cleavage plane above the SCM muscle is exposed. Along the medial side of the SCM a tunnel to the spine is created using blunt digital dissection while keeping the carotid sheath ipsilateral. In case of soft tissue resistance, sharp dissection with a blunt Metzenbaum scissor is necessary. In some circumstances, the omohyoid muscle is bulky, requiring transection to improve vision. The SCM muscle and carotid artery are retracted ipsilaterally, while the trachea and esophagus are retracted contralaterally. Next the prevertebral fascia is dissected in a craniocaudal direction using two Kocher clamps with peanut gauze. After verification of the appropriate level with intraoperative radiographs (Fig. 3), the longus colli muscle is partially detached from the vertebrae with bipolar electrocautery at the disk level. Self-retaining retractors are placed in four directions, two underneath the left and the right longus colli muscle to prevent sympathetic plexus damage. The other two retractors are placed in a craniocaudal direction to obtain a safe exposure corridor.

**Fig. 4:**
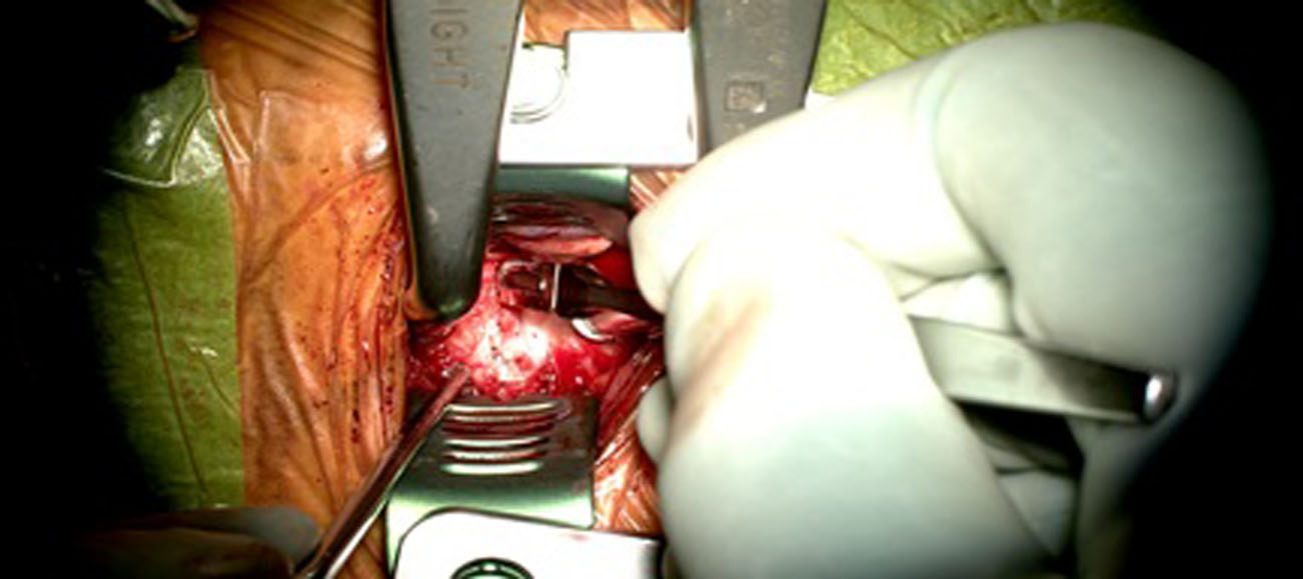
After the appropriate level has been identified, the anterior longitudinal ligament is incised

Next, the microscope is used for magnification and focused lighting to improve visualization. Before discectomy, the ALL is incised (Fig. 4). Under fluoroscopy, Caspar distraction pins are placed in the midline of the upper and lower vertebrae, and gentle distraction is applied. The upper and lower endplates are identified, and the total disk can be easily detached from the endplates using a small periosteal elevator until the posterior longitudinal ligament (PLL) has been identified.

Once the PLL has been identified, a small sharp hook is used to create a corridor to the epidural space. Then, a 1-mm Kerrison punch is used to totally remove the PLL and expose the dura. In case of large posterior osteophytes, a 4-mm side-cutting high-speed drill is used to remove the osteophytes and uncinated process. Decompression of the dura is considered appropriate if pulsations of the dura are visible. Next, a 2-mm Kerrison punch is used to decompress the neuroforamen. When a blunt tip nerve hook with at least 1 mm diameter can be freely introduced into the neuroforamen, the decompression is considered sufficient. Bleeding from the cervical epidural venous plexus can be stopped by injecting saline into the lateral recess [5] or by transiently releasing the Caspar distractor.

**Fig. 5:**
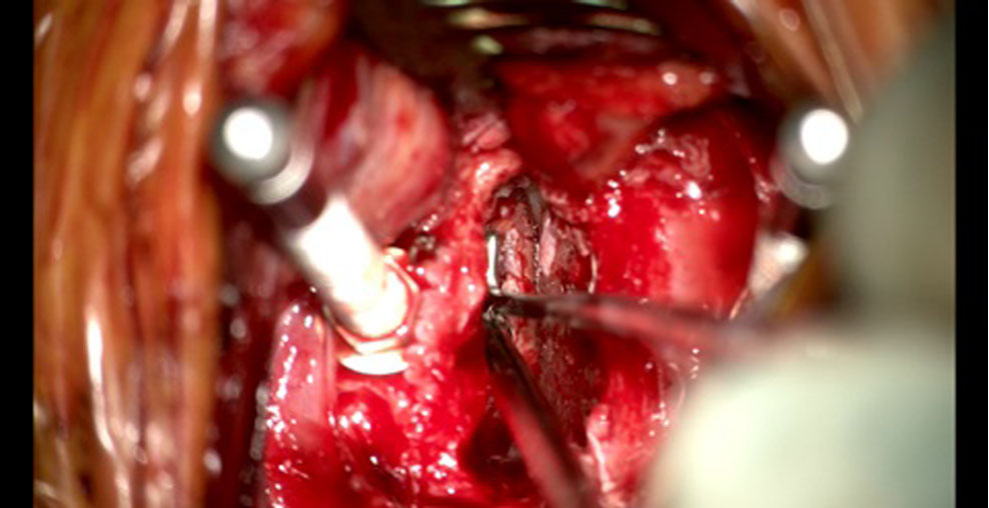
Introduction of the nerve hook into the neuroforamen

Once decompression is sufficient, the distraction is released. Now, the neuroforamen should still be accessible with a 1-mm nerve hook. If the nerve hook cannot be introduced freely, additional decompression is necessary (Fig. 5). Afterwards, the distraction screws are removed, and bone wax is used to close the pinholes. Meticulous hemostasis should be performed, and rinsing of the wound is performed until no bleeding is observed anymore. The wound is closed in three layers: the platysma, subcutis and cutis. The authors prefer to not use a drain routinely.

## Indications

Cervical radiculopathy (CR) is a common diagnosis. The most important symptom is pain, radiating from the neck to the arm. Other symptoms may include sensory loss, loss of motor function or tendon-reflex changes in the affected nerve-root distribution [2]. CR is often self-limiting and can be resolved with non-surgical treatments. Conservative treatment is recommended for at least 2 months. When conservative treatment fails and symptoms persist or increase in severity, surgical treatment is considered. Absolute indications for early surgery include progressive neurological deficit.

## Limitations

There are no absolute contraindications to ACD. A recent study revealed that ACD was expected to give a higher risk for recurrent CR compared to ACD with fusion (ACDF) [3]. Another study concluded that patients who underwent ACD had lower rates of mechanical, device-related complications, lower readmission rates, lower reoperation rates and reduced total costs than those treated with ACDF [8]. Whether fusion is necessary remains a subject for debate.

## How to avoid complications

Optimal knowledge and identification of anatomical structures are important to avoid complications. Intraoperative imaging by fluoroscopy is also recommended.

## Specific information to give the patient about surgery and potential risks

Six hours after surgery patients are allowed to mobilize. We do not advise physiotherapy, but we do advise unsupervised exercises to strengthen the extensor muscles. Patients are usually discharged 1 day postoperatively. A stiff collar may be helpful to some patients who experience neck pain. Work and daily activities should be resumed as soon as possible. Patients should build up their activities guided by the pain. Patients are scheduled to be monitored 6 weeks after the surgery at the outpatient clinic.

Commonly reported complications are:

- Dysphagia due to esophageal retraction and intubation. This complication is often self-limiting.
- Vocal cord paralysis causing hoarseness due to RLN injury. Since the left RLN loops at a lower level than the right RLN, the risk of injury to the RLN is higher in a right-sided approach [10].
- Incidental dural tears with cerebrospinal fluid leak [6].
- C5 palsy [9].

The severity of most of these complications decreases over time. Other complications are esophageal perforation, wound infection, injury of the vertebral artery, and spinal cord and nerve root injury. Rare complications include postoperative hematoma and laryngopharyngeal edema leading to airway compromise [7]. Damage to the sympathetic chain may cause ipsilateral miosis, ptosis and anhidrosis, also know as Horner’s syndrome.

## Key points

- With an adequate clinical indication and surgery performed properly, the results of this procedure are excellent.
- Routine use of intraoperative neuromonitoring is not recommended by the authors.
- Surgery from the contralateral side provides a better view of the neuroforamen.
- A false cleavage plane complicates the procedure enormously.
- Placing the tissue retractor underneath the longus colli muscle is key to preventing damage to the sympathetic chain.
- After a skin incision has been made in the front of the neck, only one thin vestigial muscle needs to be cut, after which the anatomic planes can be followed right down to the spine. The limited amount of muscle division or dissection helps to limit postoperative pain following the spinal surgery.
- With meticulous hemostasis a wound drain is not necessary.
- Appropriate dissection is associated with less than 50 ml blood loss.
- When performed properly, ACD is simply more cost-effective compared to ACDF.
- Transient dysphagia is the most commonly seen complication.

## Electronic supplementary material

ESM 1 (MP4 207946 kb)

## Abbreviations

CDH: cervical disk herniation
CR: cervical radiculopathy
SCM: sternocleidomasteoid muscle
ALL: anterior longitudinal ligament
PLL: posterior longitudinal ligament
